# Targeting ferroptosis-based cancer therapy using nanomaterials: strategies and applications

**DOI:** 10.7150/thno.65480

**Published:** 2021-10-22

**Authors:** Lianxiang Luo, Han Wang, Wen Tian, Xiaoling Li, Zheng Zhu, Riming Huang, Hui Luo

**Affiliations:** 1 The Marine Biomedical Research Institute, Guangdong Medical University, Zhanjiang, Guangdong, 524023, China.; 2 The Marine Biomedical Research Institute of Guangdong Zhanjiang, Zhanjiang, Guangdong, 524023, China.; 3 Southern Marine Science and Engineering Guangdong Laboratory (Zhanjiang), Zhanjiang, Guangdong, 524023, China.; 4 The First Clinical College, Guangdong Medical University, Zhanjiang, Guangdong, 524023, China.; 5 Experimental Animal Center, Guangdong Medical University, Zhanjiang, Guangdong, 524023, China.; 6 Affiliations Department of Medicine, Brigham and Women's Hospital, Harvard Medical School, 75 Francis Street, Boston, MA 02115, USA.; 7 Guangdong Provincial Key Laboratory of Food Quality and Safety, College of Food Science, South China Agricultural University, Guangzhou, 510642, China.

**Keywords:** Ferroptosis, Nanomaterials, Cancer therapy, Tumor microenvironment, Clinical strategy

## Abstract

As an iron-dependent mode of programmed cell death induced by lipid peroxidation, ferroptosis plays an important role in cancer therapy. The metabolic reprogramming in tumor microenvironment allows the possibility of targeting ferroptosis in cancer treatment. Recent studies reveal that nanomaterials targeting ferroptosis have prospects for the development of new cancer treatments. However, the design ideas of nanomaterials targeting ferroptosis sometimes vary. Therefore, in addition to the need for a systematic summary of these ideas, new ideas and insights are needed to make possible the construction of nanomaterials for effectively targeting this cell death pathway. At the same time, further optimization of nanomaterials design is required to make them appropriate for clinical treatment. In this context, we summarize this cross-cutting research area covering from the known mechanism of ferroptosis to providing feasible ideas for nanomaterials design as well as their clinical application. We aim to provide new insights and enlightenment for the next step in developing new nanomaterials for cancer treatment.

## 1. Introduction

Ferroptosis is a type of programmed cell death mediated by iron-dependent peroxidation proposed by Dixon* et al*. in 2012 1. Although its mechanism has not been fully elucidated, the collapse of cell membranes caused by overwhelming peroxidation is considered as a key step in ferroptosis. This process is mediated by an iron-dependent Fenton-like reaction 2. The occurrence of this overwhelming lipid peroxidation alters to some extent the morphological structure of the cell and represents a signature of ferroptosis distinct from those of other types of programmed cell death. The most remarkable difference between ferroptosis and other types of programmed cell death lies in the changes in mitochondrial morphology. The specific manifestations are shrinkage of mitochondria and disappearance of cristae under the electron microscope. Biochemically, on the other hand, it is mainly characterized by the absence of intracellular antioxidant systems, the increase of labile iron, and the enrichment of lipid substrates. Ferroptosis can be induced by various small molecule compounds referred to asferroptosis inducers (FINs) 3, and even some approved chemotherapeutic drugs have been found to induce ferroptosis.

From the perspective of cellular metabolism, it can be concluded that ferroptosis is closely associated with cellular metabolism 4. Given that ferroptosis is an iron-dependent process 2, regulating iron metabolism and/or lipid metabolism affects the sensitivity of cells to ferroptosis. At the same time, the composition of the antioxidant system depends on amino acid metabolism and the mevalonate pathway, as well as on the uptake of trace elements. Thus, the targets of ferroptosis are abundant and diverse. However, it is usually difficult to achieve adequate efficacy in the treatment of patients with small molecule compounds, which is why most drug clinical trials to date have failed. Therefore, the development of effective nanomaterials carriers is critical to improve the delivery, release, and targeting efficiency of drugs. The strategy of using nanomaterials for the delivery of drugs targeting ferroptosis has been widely studied in recent years. However, little work has been done to systematically examine the targeting mechanisms of ferroptosis, as well as ideas for constructing related nanomaterials. In this review, the mechanism of ferroptosis and various ideas for drug design and development are examined, and innovative possibilities are proposed in order to summarize previous findings and expand the horizon of drug delivery systems.

## 2. The trilogy of inducing ferroptosis in cancer

**Figure 1:**
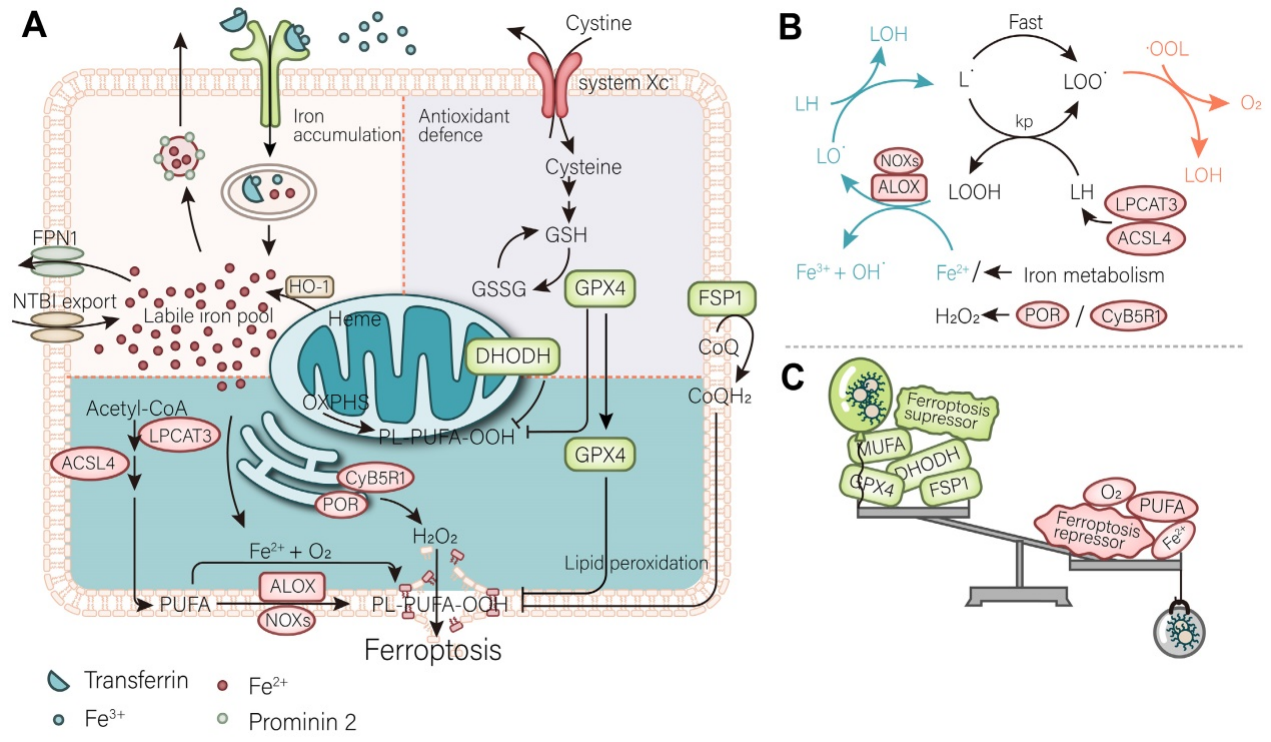
** Mechanism of ferroptosis. (A)** A mutually independent antioxidant defense axis composed of xCT-GPX4, FSP1, DHODH, protects cells against ferroptosis by antagonizing lipid peroxidation. At the same time, the entry and exit of intracellular iron as well as changes in availability regulate the sensitivity of cancer cells to ferroptosis. Lipid metabolism and PUFA synthesis provide substrates for the occurrence of ferroptosis. A series of oxidases become promoters of lipid peroxidation and the Fenton reaction. **(B)** An illustration of the role of these proteins in the detailed mechanism of lipid peroxidation is partially shown. **(C)** In general, the imbalance of intracellular ferroptosis-inducing and -inhibiting factors leads to ferroptosis, while the purpose of nanomaterials is to increase the weight of ferroptosis-inducing factors and reduce that of ferroptosis-inhibiting factors (such as antioxidant defenses).

The process of ferroptosis in cells involves three key steps: depletion of antioxidant defenses, accumulation of iron, and lipid peroxidation. The accumulation of iron leads to the continuous induction of lipid peroxidation 5. In the absence of antioxidant systems to scavenge reactive oxygen species (ROS) and protect membrane lipid, overwhelming lipid peroxidation leads to the collapse of the cell membrane, and eventually cell death. Accordingly, understanding the process of ferroptosis can help researchers better develop drugs as well as cancer treatment strategies (Figure 1).

### 2.1. The depletion of antioxidant defense

To date, three antioxidant defense systems, namely GPX4, FSP1, and DHODH, which localize to different parts of the cell and function independent of each other, have also been found to play a complementary role in inhibiting ferroptosis. Since each antioxidant defense system is independent, their status needs to be considered comprehensively according to the cancer type when developing drug and clinical treatment strategies.

GPX4, which localizes in the cytoplasm, is generally considered the predominant ferroptosis resistance factor 1, 6. It forms an antioxidant defense system with the membrane protein system xc^-^. GPX4 is a selenoprotein whose active site contains a selenocysteine 7, which converts toxic lipid peroxides (L-OOH) into a non-toxic lipid (L-OH) form by catalyzing them, thereby curbing continuous lipid peroxidation. GPX4 is indispensable to perform this function, together with its substrate GSH 8, 9. During peroxidation, GSH is converted into its oxidized form GSSG and regenerated into GSH by GSH-disulfide reductase (GSR) using flavin adenine dinucleotide (FAD) as coennezyme and reduced nicotinamide adenine dinucleotide phosphate (NADPH) as cofactor, which continuously provides power for GPX4. Conceivably, the abundance of GSH determines the catalytic efficiency of GPX4 and the sensitivity of cancer cells to ferroptosis. Downregulation of GSH has been observed in cells undergoing ferroptosis. The rate-limiting step in the synthesis of GSH is the ligation of cysteine to glutamate, which is catalyzed by GCLC. In contrast, the extracellular intake of cystine, a precursor of cysteine, is the most important source of intracellular cysteine 10. System xc^-^ is a key cystine-glutamate transporter, consisting of two subunits, namely SLC7A11 and SLC3A2 11, which transports cystine into the cell in exchange for glutamate in a 1:1 ratio. Since it regulates the production of GSH, its activity is essential for the anti-ferroptosis effect of GPX4. P53, an important transcription factor in cancer cells, triggers ferroptosis and suppresses cancer development by inhibiting the expression of SLC7A11 12. Conversely, Nrf2 upregulates SLC7A11 and GPX4, thereby inhibiting ferroptosis through its anti-oxidative stress activity 13.

However, it turns out that GPX4 is not the only antioxidant defense system within cancer cells. The discovery of two other known antioxidant pathways not only indicates that GPX4 is not the only ferroptosis suppressor but also partially reveals the ferroptosis resistance of some cancer cell lines to the targeted system xc^-^-GPX4 axis. Two independent laboratories simultaneously identified FSP1 as an antioxidant system localized on the cell membrane 14, 15. FSP1 exerts its activity as an antioxidant defense system parallel to that of the GPX4 system. In addition to being a member of the electron transport chain, CoQ10 also plays a role in ferroptosis. Its reducing mode, ubiquitin, can inhibit the peroxidation of lipids and suppress ferroptosis. However, FSP1 suppresses ferroptosis by catalyzing the continuous regeneration of CoQ10 14, 15. It is well established that the peroxidation of mitochondria and cell membranes is the initial step of ferroptosis. Since there is an antioxidant system specialized in cell membranes, there is naturally an antioxidant system specialized in mitochondria. DHODH is an antioxidant defense system recently identified in mitochondria, where it localizes to the inner mitochondrial membrane 16. DHODH indirectly promotes the regeneration of CoQH2 by reducing FMN and inhibits lipid peroxidation in mitochondria. The increase in DHODH activity increased the resistance of cancer cells to ferroptosis in cell lines overexpressing GPX4 and FSP1.

Current studies on ferroptosis-targeted cancer therapy have focused on antioxidant defenses, particularly the system xc^-^ - GPX4 axis. Available clinical drugs, such as erastin and sorafenib, have been shown to inhibit system xc^-^ 17, 18. Alternatively, FIN-like compounds (e.g., RSL3) are the most widely used GPX4 inhibitors to significantly induce ferroptosis. However, targeting the other two systems in parallel to GPX4 cannot be overlooked. It has been experimentally demonstrated that iFSP, an inhibitor of FSP1, and BQR, a DHODH inhibitor, can reverse part of the resistance to ferroptosis in targeted GPX4-tolerant cell lines. Therefore, these pathways should be considered comprehensively in future drug development. Personal consideration of the patient's phenotype for personalized targeted therapy is required in theranostics.

### 2.2. The accumulation of iron

Accumulation of iron is a prerequisite for ferroptosis. Ferroptosis is triggered by divalent iron ions, whose abundance determines the sensitivity of cells to ferroptosis. Instead, only free iron can participate in the triggering of ferroptosis (which will be discussed in the following subsection) 2. In this subsesction, we describe various ways in which the abundance of divalent iron ions in cells is affected. Both exogenous input of iron and intracellular iron have value for use with targeted nanomaterials.

Transferrin is a protein that binds extracellular ferric ions and is transported intracellularly by binding to TfR on cancer cells 19, 20. The ferric ions released from transferrin are subsequently reduced to ferrous ions that drain into the labile iron pool (LIP). The TfR is upregulated during ferroptosis, thus it is recognized as a biomarker of ferroptosis 21. In fact, due to their metabolic peculiarity, cancer cells themselves have a higher iron requirement than normal tissues. Therefore, the expression level of TfR is generally maintained at a relatively high level in cancer cells 22. These properties enable the possibility to induce ferroptosis in cancer cells and suggest that TfR can be used as a specific target for enrichment of nanomaterials. In addition, several membrane proteins (SLC39A14, SLC11A2, SLC39A8) that mediate non-transferrin-bound iron (NTBI) 23 trafficking also mediate iron ion entry and increase the LIP and, together with the TfR, control the import of exogenous iron to regulate ferroptosis 24. Ferritin controls the size of the LIP by binding free iron. Ferritinophagy is a ferritin-specific type of autophagy that is mainly triggered by NCOA4 and mediates the degradation of ferritin, releasing free iron to expand the LIP 25. Undoubtedly, ferritinophagy enhances cellular sensitivity to ferroptosis and serves as a key way of regulating ferroptosis. In mitochondria, heme oxygenease 1 (HMOX1) breaks down heme to release free iron 26. Meanwhile, FXN regulates ferroptosis sensitivity by limiting iron availability by synthesising iron-sulfur clusters. Downregulation of FXN has been found to sensitize cancer cells to ferroptosis 27. In contrast, iron export decreases the LIP and sensitizes cancer cells to ferroptosis. Ferroportin 1 (FPN1, also known as SLC40A1), is the only known iron transporter mediating iron transport from the inside of the cell to the outside of the cell in humans. High expression of FPN1 can sensitize cells to ferroptosis 28. At the same time, prominin 2 mediates the formation of ferritin-containing vesicular bodies and exosomes that transport iron out of the cell by exocytosis, similarly reducing the sensitivity of cancer cells to ferroptosis 29.

Measurement of the levels of iron, *in vitro,* has confirmed that ferroptosis occurs when iron overload occurs, while measurement of *in vivo* levels have also confirmed that a high iron diet can increase the efficiency of cancer therapy targeting ferroptosis. This has led to the idea of developing nanomaterials to regulate ferroptosis based on iron overload. While development of drugs targeting ferritinophagy is worth tapping into, negative regulators targeting ferritinophagy increase intracellular free iron levels.

### 2.3. The occurrence of lipid peroxidation

The ultimate effect of free iron accumulation is to induce lipid peroxidation. In general, the Fenton-like reaction of ferrous ions with membrane phospholipids is the beginning of the entire lipid peroxidation chain reaction. On the one hand, polyunsaturated fatty acids (PUFAs) are excellent substrates for this reaction because the C-H bonds of the methylene groups on either side of the C-C double bond are the weakest known C-H bonds (about 76 kcal/mol). Arachidonic acid (C20:4) and epinephrine (C22:4), meanwhile, are considered the most predominant reaction substrates and are enriched at the beginning of ferroptosis. Iron and oxygen are involved in the Fenton reaction in lipid peroxidation to generate hydroxyl radical, seizing hydrogen atoms from the substrate to generate carbon-centered radicals, then react with O_2_ to generate peroxyl groups, through the peroxyl group generated through the reaction with another molecule of substrate, continue to perform Fenton-like reaction with divalent iron to create the chain reaction (Figure 1). The termination reaction competes with this process to generate carbonyl compounds, alcohols, and oxygen. Hydrogen peroxide and other oxidases (ALOXs, NOXs) can also initiate the above reaction by producing peroxyl groups 30, 31. POR and CYB5R1, which located in the endoplasmic reticulum (ER), are essential sources of hydrogen peroxide and have been shown to induce ferroptosis 32. On the other hand, monounsaturated fatty acids (MUFAs) and ether phospholipids block lipid peroxidation to prevent ferroptosis 33, 34. Regulation of membrane phospholipid synthesis pathways regulates sensitivity to ferroptosis. ACSL4 with LPCAT3 is a key regulatory enzyme in the formation of PUFA phospholipids, while ACSL4 is enriched during ferroptosis and can serve as a biomarker of ferroptosis 35.Instead, ACSL3, a key enzyme catalyzing MUFA biosynthesis, is a negative regulator of ferroptosis 33.

Recently, it has been reported that beneficial small-molecule oxidants trigger ferroptosis by direct oxidation of lipids 36, which can be combined with nanocarriers to enable their precise targeted delivery to tumour tissues only. Therapeutic strategies that additionally induce the production of oxidants within cancer cells, such as hydrogen peroxide, are also feasible. They can additionally make it more suitable for targeted ferroptosis therapy by promoting the enrichment of PUFAs within cancer tissues.

## 3. Ideas for the development of nanomaterials to target ferroptosis

The nature of the nanocarrier itself and the active ingredients it carries, its applicability to cancer, and type of patient determine the quality of the clinical treatment. The development strategies of drugs targeting ferroptosis are therefore particularly important. Besides targeting strategies that trigger ferroptosis directly, synergy with radiation therapy and immunotherapy also needs to be considered.

### 3.1. Targeting antioxidant defense and lipid peroxidation

**Figure 2:**
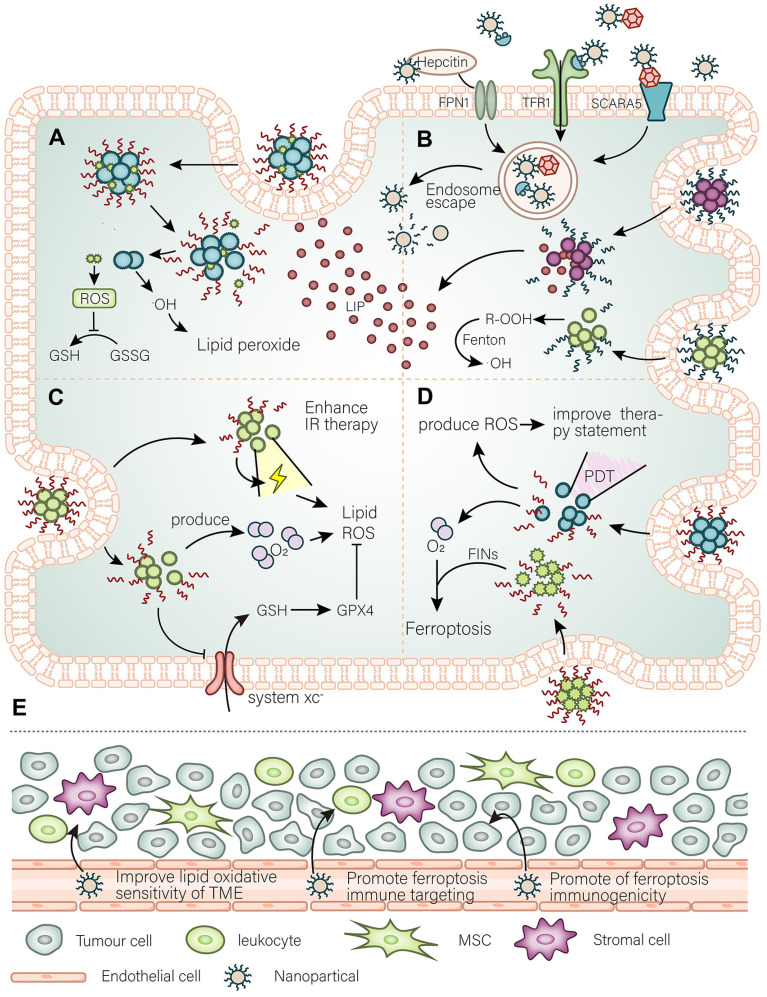
** The main purpose of nanomaterials to target ferroptosis. (A)** A general simple strategy for the development of nanomaterials is to improve the targeting of ferroptosis inhibitors to cancer tissues and modify the intracellular oxidative environment. **(B)** At the same time, the high demand for iron by cancer cells is the basis for the development of nanomaterials to target cancer cells, such as using Tfr to further expand or utilize the abundant unstable iron pool in the cancer cells. **(C)** By developing a strategy for releasing O_2_ as well as weakening of antioxidant defense systems to promote the damaging effect of ionizing radiation (IR) and further promote IR-induced ferroptosis. **(D)** Photodynamic triggered nanomaterials improve the treatment quality by adding an oxygen-releasing strategy combined with FINs. **(E)** In the TME, nanomaterials promote cancer cell immunogenic ferroptosis, promote infiltration of immune cells, and adjust the lipid balance of the immune microenvironment to curb the invasion and spread of cancer cells.

Breaking down antioxidant defenses means breaking down the defense that cancer cells have for protection against ferroptosis. However, some types of cancer cells possess an active antioxidant defense system, which renders them resistant to ferroptosis. The impairment of the antioxidant defense system sensitizes cancer cells to ferroptosis (Figure 2). There have been many studies on the induction of ferroptosis in cancer cells by small molecules against antioxidant defense system targets. For example, the traditional anticancer drug sorafenib targets system xc^-^ in solid tumors and induces ferroptosis 17. In addition, sulfasalazine was demonstrated to target ferroptosis in prostate cancer, and lymphoma 37, 38. In experimental studies, the well-characterized erastin 39, RSL3 40, iFSP, and BQR have been shown to target various antioxidant defense systems leading to ferroptosis. It is worth mentioning that traditional Chinese medicines, such as artemisinin and its derivatives, have good effect against antioxidant activity and deserve to be considered as drug development candidates. To develop nanomaterials against an antioxidant defense system, it is necessary to comprehensively consider the parallel antioxidant defense systems and select compounds with good selectivity and kinetics. Given the heterogeneity of tumor antioxidant defense systems in different patients, their suitable nanomaterials can be ascertained by clinical diagnosis for precise personalized medical treatment.

There are now various cases of ferroptosis inducers loaded in nanomaterials. However, there are still some ideas that can be developed into effective ferroptosis-based therapy for cancer. Sulfur dioxide can regulate the balance of oxidative levels in tumors, and For instance, Shen *et al*. 41 developed a GSH-responsive sulfur dioxide polymer prodrug for use as a carrier for the corresponding loaded drugs. This carrier cleverly uses intracellular GSH (thiolyl) as its triggering molecule and rapidly releases sulfur dioxide from N-(3-azidopropyl)-2,4-dinitrobenzenesulfonamide (AP-DNS), a drug external to the nanocarrier, leading to an increase in intracellular ROS and a decrease in GSH. This type of carrier can be simultaneously used as a prodrug for multidrug-resistant tumors and as a prodrug for photodynamic therapy (PDT) 42, as described below. These findings demonstrate the potential for using this nanocarrier to carry ferroptosis inducers to treat tumors. Another multistage synergistic nanoplatform that achieves co-delivery of proteins with drugs is equally noteworthy. In the initial study by Zhang *et al*. 43, they chose an exquisitely designed three-piece copolymer, namely mPEGb-PGCA-b-PGTA. The mPEGb-PGCA-b-PGTA copolymer can achieve endosomal escape and drug release triggered by pH changes. Zhang *et al*. used this copolymer to carry the chemotherapeutic drug DOX with modified RNAse to induce intracellular ROS and RNA breakdown and successfully kill cancer cells. The advantage of this nanoplatform is its rich plasticity, and the expectation is to carry a variety of ferroptosis inducers to target different antioxidant defense systems and carry oxidase (or regulate peroxide production enzyme) proteins to induce ferroptosis in cancer cells by synergy. In sum, selecting the correct carrier and comprehensively considering the different antioxidant defense systems within cancer cells is the key to the development of nanomaterials to effectively target ferroptosis in cancer cells.

PDT, a therapy that uses light and photosensitizers, kills cancer cells through light-triggered chemical damage. As a popular treatment, PDT has been used in combination with nanomaterials therapy. Simultaneously, PDT can be combined with other traditional treatment modalities 44. Most notably, PDT can increase the immunogenicity of tumors. PDT can induce cell death through a variety of cell deathtypes, and the process can be artificially guided. Due to the different properties of PCDs (programmed cell deaths), guided ferroptosis may be the best choice for cancer treatment. There have been some ingeniously conceived nano-mediated PDT approaches targeting ferroptosis (introduced in the next section). The most significant disadvantage of PDT-based strategies targeting ferroptosis is the oxygen deficiency in tumors, but such disadvantage can be circumvented. The general idea is to add oxygen to the donor or induce oxygen-independent free radical generation. In a study by Xu* et al*. 45, they combined hemoglobin with photodynamic nanomaterials. On the one hand, hemoglobin provides oxygen for PDT, and on the other hand essential iron for ferroptosis. In addition, Zou *et al*. 46 developed a simple oxygen release strategy to achieve chemical energy storage, which can be used to continuously release oxygen in a dark hypoxic tumor environment after laser triggering. Also, the BiOI@Bi2S3@BSA nanoparticles (NPs) constructed by Zhao* et al*. 47 can produce oxygen free radicals based on electron-hole pairs through a PDT process using X-ray irradiation, providing an additional oxygen-independent approach. In summary, besides solving the problem of hypoxia, the use of PDT for the guidance of ferroptosis in treatments targeting ferroptosis deserves to be given attention. Meanwhile, the use of PDT should take into account the directionality of ferroptosis.

### 3.2. Targeting iron metabolism and availability

Cancer cells meet their demand for iron by enhancing iron metabolism. Thus, on the one hand, sufficient iron enables to meet the need for vigorous biological activity of cancer cells, while on the other hand abundant iron has the potential to induce ferroptosis in cancer cells. Predictably, cancer cells upregulate various membrane proteins that control various forms of iron entry to meet their iron requirement. A variety of membrane proteins such as TfR1, FPN, SCARA5, and CD163 are upregulated in cancer cells and become possible targeting candidates. There have been several nanomaterials that target tumor cells through use of the TfR1 (NCT02340117, NCT00964080, NCT02340156). In addition, worth noting is a nanomaterial loaded with artemisinin recently reported 48, which also uses the TfR1 to target tumors, while artemisinin is loaded into fullerenes and modified with hyaluronic acid. These nanomaterials not only have good targeting ability but also are suitable for PDT. Moreover, compared to other nanomaterials, nanomaterials that use transferrin also have the ability of penetrating the blood-brain barrier and can be used to perform targeted therapy to such brain tumors. Since transferrin can cross the blood-brain barrier to deliver iron to the central nervous system and meet its demand for iron, compared to other nanomaterials, transferrin-containing NPs also have the ability to penetrate the blood-brain barrier and deliver drug molecules into the central nervous system, and thus can be used to perform targeted therapy for central nervous system brain tumors. Therefore, ferritin is also an excellent choice for the development of nanomaterials for use in targeted cancer therapy. Ferritin receptors are upregulated in cancer. In addition, the structural and physicochemical characteristics of ferritin make it suitable as a drug carrier. For instance, ferritin can form a unique nanocage structure 49, which can carry a large number of drug molecules. Moreover, the formation of this structure is pH-sensitive 50, decomposes at lower pH, and assembles at physiological pH. There are already therapeutic strategies that use this structure to target cancers, such as pancreatic cancer 50. It is also relatively easy to fabricate ferritin nanomaterials to target ferroptosis in cancer cells. Hepcidin is a class of factors that regulate the systemic iron cycle and mediate its own endocytosis together with FPN. Also, the abundance of hepcidin itself* in vivo* can directly regulate ferroptosis 51. Therefore, it is likely that the use of hepcidin for targeting ferroptosis is relatively efficient. Although there is no specific therapeutic strategy, drug development targeting the endocytosis of hepcidin and FPN to induce ferroptosis, it is worth a try. When implementing a particular clinical treatment, diagnostic means first should determine the target expression level of cancer cells. Given the increased iron levels in cancer cells, therapeutic strategies targeting unstable iron pools are feasible. Very recently, monodispersed ferrihydrite NPs, synthesized inspired by organisms that naturally synthesize ferrihydrite biominerals, were reported to release enormous amounts of free Fe^2+^ and mediate related lipid ROS production after being excited by blue light 52. The advantage of this approach is that it overcomes the *in vivo* toxicity in humans caused by the vast majority of Fe^2+^ releasing particles. Similarly, only the LIP present within cancer cells can be used to induce ferroptosis to kill cancer cells without the need to consider toxicity issues—co-loaded NPs of peroxide (R 'OOH) and the ferroptosis inducer erastin 53. R'OOH can initiate the Fenton reaction with the LIP in a rapid response and further induce lipid peroxidation. In developing such endogenous nanomaterials, targeting intracellular iron metabolism (e.g., promoting ferritinophagy) to release free iron ions to expand the endogenous LIP can be considered. It is a good option for ferritin and mitochondrial iron. This study expands the idea of using the Fenton reaction to treat cancer. Arachidonic acid and adrenal acid, the best substrates for ferroptosis, can be peroxidized and targeted enriched in cancer cells using nanomaterials as "kindlings" triggered by ferroptosis. This idea is inspired in the reasons for a similar iron overload that has been preliminarily confirmed in a recent study 54. In addition, the scheme can synergistically weaken the antioxidant defense systems. Adequate understanding of the status of iron in cancer is important to develop precisely targeted ferroptosis nanomaterials, which must take into account both the peculiarity of iron metabolism in cancer cells and the iron requirement in normal tissues (Figure 2).

### 3.3. Potential strategies for collaborative radiation therapy

Radiation therapy can lead to various cellular phenomena, such as activation of ataxia-telangiectasia mutated (ATM), and p53, which promotes cell death. Recent studies have confirmed the relationship between radiation therapy and ferroptosis 3, 55. Radiation therapy induces ferroptosis in cancer cells through multiple pathways. Through the generation of oxygen free radicals, radiation therapy also disrupts the redox balance of cells by reducing GSH, SLC7A11 56, and reducing antioxidant defenses *via* ATM 57 and p53. In addition, radiation therapy induces a range of cellular effects, such as a variety of autophagic behaviors that promote ferroptosis and ACSL4 production 58. Meanwhile, cells resistant to radiotherapy usually show resistance to ferroptosis, and the hypoxia-associated HIF pathway is activated in these cells. The hypoxic tumor microenvironment (TME) represents an essential mechanism of radiation resistance. Activation of the HIF pathway makes cancer cells resistant to ferroptosis, which on the other hand, enhances oxidative stress in cells. Therefore, radioresistant cells are highly dependent on antioxidant defense systems and can be used as a breakthrough. There are already various small molecule drugs against antioxidant defense systems that increase the effect of radiotherapy *in vivo* 59. The same function of nanomaterials is not reproduced here, but in cells resistant to radiotherapy, resistance to ferroptosis is observed and the hypoxia-associated HIF pathway is activated 60. Furthermore, cancer cells achieve radioresistance by reprogramming lipid metabolism. For example, KRAS mutant cancer cells upregulate ACSL3 to enrich MUFA, which makes it difficult to trigger ferroptosis, thereby achieving radioresistance 61. Thus, it is possible to develop nanomaterials to enrich phosholipid-bound-PUFA (PL-PUFA) in the tumor environment and effectively cause radiosensitization (Figure 2).

In addition, a variety of radiosensitizing agents have been developed to enhance therapeutic efficacy and reduce radiation toxicity to normal tissues. Due to their physicochemical properties, nanomaterials with inorganic configurations seem to be the optimal choice for radiosensitization. Such nanomaterials usually allow cancer cells to rapidly produce ROS under radiotherapy conditions and can create a good condition to induce ferroptosis. The targeting of nanomaterials, synergy with other therapies, and timely clearance in patients need to be considered for their development. For example, a selective Cu_2_(OH)PO_4_ nanocrystals can catalyze the degradation of hydrogen peroxide into hydroxyl radicals by the corresponding X-ray stimulation, and the process can only be performed in the hypoxic TME 62. Meanwhile, since the reaction cannot proceed in oxygen-rich normal tissues normal cells are not harmed. In summary, the combination of nano-induced ferroptosis with radiotherapy requires careful consideration of the substrates of ferroptosis, the tumor oxygen-poor environment, and strategies to target antioxidant defense systems. Targeted nano prodrugs can be developed to regulate the lipid metabolic balance of the TME, before radiotherapy, rendering them suitable for radiotherapy targeting ferroptosis, thereby preventing further development of cancer, and improving prognosis.

### 3.4. Possible strategies for synergistic immunotherapy

The relationship between ferroptosis and immunity is partially understood. The role of immunotherapy against PD-L1/PD-1 in ferroptosis has been explained in part. CD8 + T cells have been found to act on cancer cells by releasing INFg, leading to the downregulation of antioxidant defense systems and ultimately inducing ferroptosis 63. Also, treatment with anti-PD-L1 greatly enhanced the killing efficacy of CD8 + T cells against cancer cell-induced ferroptosis. Moreover, therapies against PD-L1 in cells resistant to ferroptosis showed a resistance effect 63. Accordingly, the focus of the development of drugs targeting ferroptosis should be set on: (1) remodeling the sensitivity of cancer cells to ferroptosis; (2) enhancing the synergy of the immune response and PD-L1; (3) increasing the activity of T cells and intratumoral penetration. The way cancer cells regain their sensitivity to ferroptosis has been described above. However, attention needs to be paid to the selectivity of nanomaterials for targeting cancer cells to avoid causing unnecessary death of immune cells. The approach to achieve synergistic anti-PD-L1 immunotherapy has been developed. For example, the nano platform developed by Li *et al*., which can enlarge LIP and enhance the function of CD8 + T cells and their intratumoral infiltration as well as synergize with immunotherapy 64.

Ferroptosis is also regulated by other immune activities. For example, we now know that ferroptosis is immunogenic. Damage-associated molecular patterns (DAMPs), PGE2, and other substances released by ferroptotic cells can inhibit or activate immune cells 65, although it remains unclear whether ferroptotic cells will release specific substances to regulate the immune system. Also, oxidized phospholipids on the cell membrane act as "eat me" signals 66 when ferroptosis sets off and recruits phagocytes. Since the EMT status of cells is highly sensitive to ferroptosis, the tumor microenvironment becomes the key to their migration success rate. The abundance of different iron and fatty acid species in the immune microenvironment determines the difficulty of cancer cell invasion and metastasis and regulates cancer cells' immune evasion. For example, cancer cells in the lymphatic environment are more likely to metastasize than cancer cells in the blood vessels. For this reason, the purpose of nanomaterials is to remodel the tumor immune microenvironment: sensitize to ferroptosis, prevent its metastasis, enhance the killing effect of immune cell-induced ferroptosis, improve the inhibitory effect of cancer cell ferroptosis on immune cells, and enhance the immunogenicity of ferroptosis in tumor tissues. Just recently, the nanomaterials Fe3O4-SAS @ PLT developed by Jiang* et al*., consisting of dielectric magnetic NPs (Fe3O4) loaded with sulfasalazine (SAS) and camouflaged with platelet (PLT) membranes, triggered ferroptosis by inhibiting the glutamate-cystine countertransport system xc pathway. Thus, the research to date has revealed that these nanomaterials significantly transformed TME macrophages from M2 to M1 phenotype, while having a good synergistic effect with anti-PD-L1 therapy 67. Coincidentally, another study 68 has also reported that nanomaterials targeting ferroptosis can also promote the conversion of M2 macrophaes to M1 macrophages within the TME and attenuate PD-L1 expression in the TME. Their study suggests that ferroptosis promotes antitumor immunity 68. Another study, confirmed the promotion of the recruitment of infiltrating T cells by targeted ferroptosis nanomaterials and suggested their joint promotion with immunotherapy 69. However, a better understanding of the relationship between ferroptosis and the TME is required to fully grasp this type of therapy (Figure 2).

Moreover, the differences in the sensitivity of immune cells to ferroptosis within the TME also illustrate difficulty of developing a nanotherapy targeting ferroptosis. For example, antitumor CD8 + T cells are similarly sensitive to ferroptosis 70. Compared with conventional CD4 + T cells, CD8 + T cells are more sensitive to inhibitors of GPX4. Meanwhile, the CD36 lipotranslocase expressed by CD8 + T cells was found to increase ferroptosis sensitivity and impair the function of antitumor immunity 71. Overexpression of GPX4 and treatment with ferroptosis inhibitors reversed this phenomenon. However, CD8 + T cells have a higher cysteine utilization efficiency, and they are not as sensitive to inhibitors of system xc - as cancer cells 72. Also, Tregs that are detrimental to immunity evade ferroptosis by upregulating GPX4 73. These lines of evidence illustrate the need for nanomaterials targeting ferroptosis to be selective for cells, or for strategies that provide therapeutics against different immune microenvironments. Targeting the epidemic desert or promoting the immune microenvironment of tumors can directly and highly target ferroptosis and increase the infiltration of antitumor immune cells. Aiming at the microenvironment with good immune infiltration, a nanomaterials can be developed, which is only specifically enriched in tumor cells, while sparing antitumor immune cells from significant lethal effects. Options include targeting iron metabolism-related proteins on the cell membrane surface, or using different intracellular pH to control the release of drugs. In addition, CD8 + T cells can be further targeted to evade ferroptosis, for instance, by targeting the CD36 receptor to reduce their sensitivity to ferroptosis. With the increase of the understanding of ferroptosis in tumor cells and immune cells, more "differentially targeted" strategies will be proposed.

## 4. Progress of nanomaterials in targeting ferroptosis

With the deepening of the study on the mechanism of ferroptosis, there has been good progress in the anticancer treatments with nanomaterials targeting ferroptosis. The classification of the mechanisms of action of these nanomaterials has been discussed in more detail in other reviews 74-76. Nanomaterials targeting ferroptosis are generally classified into iron-based and non-iron-based nanomaterials. In this section we will describe the current status of these two types of nanomaterials targeting ferroptosis. More representative nanomaterials are summarized in Table 1. In any case, the design objectives of these nanomedicines are described in the above two sections of this paper.

### 4.1. Iron-based nanomaterials

This type of nanomaterials has some advantages in its ability to directly trigger the Fenton reaction. As the name implies, this class of nanomaterials contains iron with the general purpose of increasing the availability of intracellular iron. This nanomaterials use the Fenton reaction to disrupt the intracellular oxidative balance. The vast majority of iron-based nanomaterials are designed to target tumors specifically and can trigger and release iron at specific times. Alternatively, the oxidative balance of cells is disrupted by the nanomaterial itself promoting the Fenton reaction.

Iron-based nanomedicines are usually classified into the following types:

Iron oxide NPs (IO NPs): is a simple nanomaterial that has been approved by the United States Food and Drug Administration (FDA) agency for the treatment of iron deficiency. The most primitive IO NPs usually simply kill cells by producing an overwhelming amount of ROS through the release of free iron. In addition, this nanomaterial can also be further expanded to achieve better ferroptosis-inducing effect or tumor targeting. In general, the purpose of this expansion is to better promote the Fenton reaction and produce ROS, or to confer imaging diagnostic function. For example, Ma *et al*. co-loaded cisplatin with a nanosized drug to overcome resistance to cisplatin and further induce ferroptosis 77. Alternatively, through modification, the release of oxygen is combined with the release of iron ions. For example, Zhou *et al*. attached IO NPs to linoleic acid hydrogen peroxide (LAHP) and produced a triggerable IO-LAPH-NPs 78. These NPs can produce singlet oxygen through the Russell reaction of Fe2 + with the LahP produced by IO NPs under acidic conditions at the tumor site. In addition, Li *et al*. further encapsulated IO NPs with H_2_O_2_ into polymers to form the structure of H_2_O_2_/fe3o4 - PLGA polymer bodies 79. The encapsulation of H_2_O_2_ plays a crucial role in simultaneously providing O_2_ for echo reflection and OH as therapeutic ROS.

Iron-doped nanomaterial, or amorphous iron NPs: these nanomedicines usually refer to nanomaterials mixed with amorphous iron. This nanomaterial usually relies on the release of its mixed iron to sensitize cells to ferroptosis and achieve better killing effects. In one study, zinc (II) protoporphyrin IX (ZnP) was injected into the particle oxyurea (BFR) to replace the heme group, extending the N-terminal portion of the BFR using peptides that could target overexpressed receptors on tumor vasculature and cells. The Fenton reaction between intracellular Fe_2_ + and H_2_O_2_ promoted by this nanomedicine targets iron-loaded ZnP-BFR structures, produces -OH and oxygen upon light irradiation, and inhibits cancer cell development 80.

Iron-organic frameworks: these nanomedicines have ultra-high porosity (up to 90% free volume) and huge inner surface regions, as well as strong extensibility 81. Thus, catalytic membranes can be formed with potential anticancer potency. In addition, this nanomaterial is pH responsive and can be further expanded, such as by the addition of anticancer small molecule components 82. These nanomedicines also have the imaging ability of magnetic resonance. In summary, these nanomedicines have great potential to be developed.

### 4.2. Non-iron-based nanomaterials

This class of nanomaterials does not contain iron and is extremely rich in diversity. The purpose of non-iron based nanomaterials is to promote the Fenton response, disrupt antioxidant defense systems, and even regulate cellular metabolism to achieve the ultimate purpose of promoting ferroptosis. These nanomaterials have the potential to carry inducers of ferroptosis to achieve better therapeutic efficiency. They also have the potential to promote the Fenton reaction by expanding PUFAs, or using intracellular LIPs. The triggering modes of these nanomaterials are also relatively diverse. Most of the PDT drugs fall into this category. In addition, nanomaterials, partially composed of other metal elements, may promote ferroptosis by disrupting the activity of iron receptors/channels on the cell membrane 83.

Non-iron-based nanomaterials are usually available in the following forms:

Simple FIN carrier system: these nanomedicines trigger ferroptosis by carrying a FIN as the main component. Among them, mPEG-PLys-AA/RSL3 releases RSL3 to trigger ferroptosis and kill cells 84. Due to the general nature of this class of drugs, these nanomedicines usually have to improve their release, response, and enrichment mechanisms to achieve more effective therapeutic effects and reduce toxicity to normal tissues.

Photodynamic/sonodynamic nanomedicines: these nanomedicines have release mechanisms that can be actively triggered and have potential for use in diagnostic applications. Some of these nanomedicines have already been mentioned in the previous section. In addition, such nanomedicines are usually expanded, such as by carrying oxygen release mechanisms to adapt to the hypoxic environment in tumors 45, 85.

Lipid enriched nanomaterials: these nanomaterials are designed to rapidly trigger ferroptosis by promoting the enrichment of PUFAs within the tumor cell/tumor microenvironment. Such strategies to trigger ferroptosis are less noticed, but are very effective. In addition, since the substrates carried are common in the human body and can be metabolized normally, such nanomedicines may have the lowest toxicity. The “kindling" strategy was mentioned above. Among this type of nanomedicines, LDL-DHA induces ferroptosis through the omega-3 fatty acids it contains 86. This nanomedicine can expand the oxygen release strategy to counteract the hypoxic environment within cancer cells that is not conducive to lipid peroxidation.

Nanodrugs carrying non-coding RNA (ncRNA): these nanomedicines weaken the ferroptosis defense mechanism within cancer cells by preventing the translation and gene expression of certain proteins involved in the defense against ferroptosis, which is lethal to cancer cells. Since there have been numerous studies on ncRNAs and ferroptosis, there are a large amount of materials for rfurther experimental research. However, compared with the treatment targeting ferroptosis with ncRNAs alone, gene interfered-ferroptosis therapy may be a better way to use ncRNAs to fight cancer, and this combined treatment strategy will be introduced in the next section.

## 5. Application and perspective in nanomaterials for targeting ferroptosis

**Figure 3:**
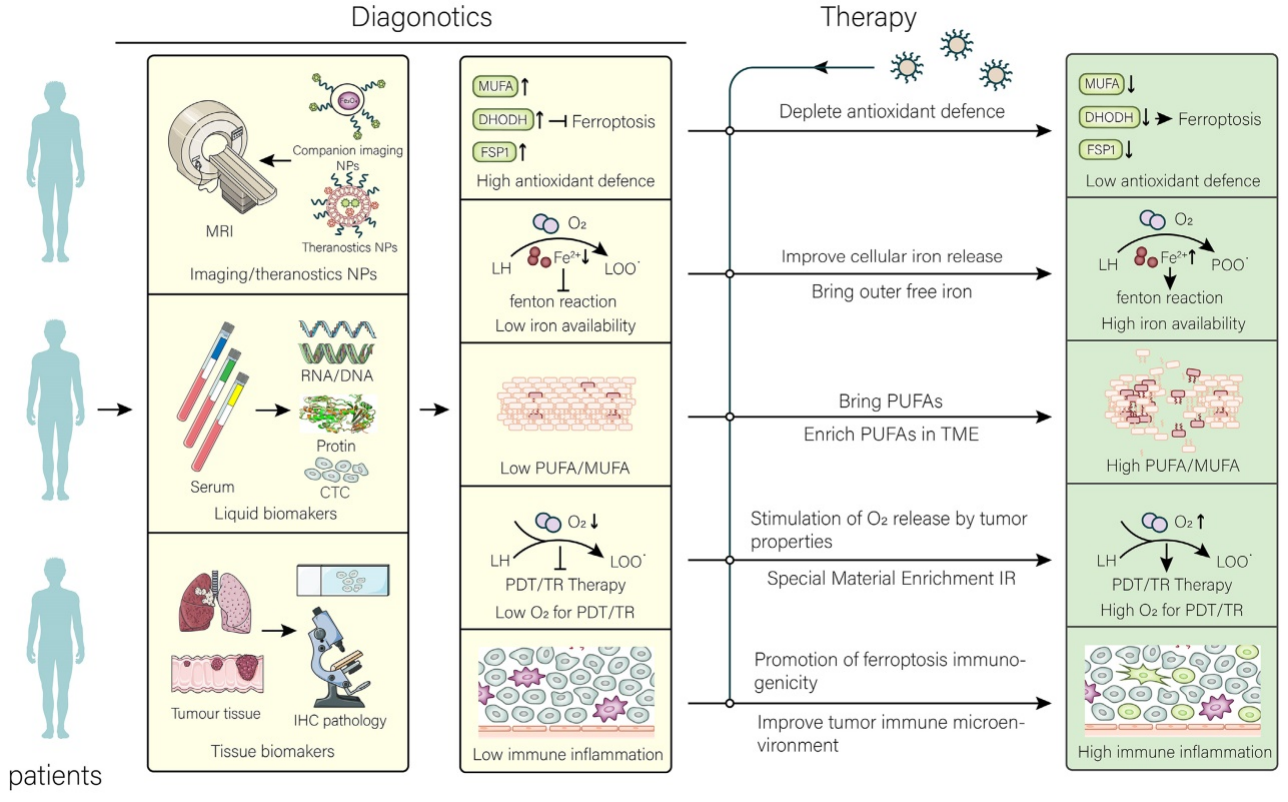
**Application and perspective in nanomaterials for targeting ferroptosis.** In the clinic, the aim of nanomaterials is to overcome the limitations of different tumors and their microenvironment. After determining the limitations that are not conducive to treatment by different diagnostic tools, the targeted treatment is performed using the corresponding nanomaterials to achieve the best efficacy at the lowest cost. The common resistance of tumors to ferroptosis has been overcome by therapeutic strategies.

The nanomaterials used for targeting ferroptosis should be precise and effective. In this section, we elaborate on the relevant diagnostic and therapeutic strategies and discuss some problems that need to be addressed (Figure 3).

### 5.1. Diagnostic strategy

The primary analytic for nanomaterials used for cancer treatment by targeting ferroptosis is the patient's enhanced permeability and retention (EPR) effect. The EPR effect affects the efficiency of nanomaterial therapy, and the effect has been well described in other reviews 118. Diagnostic nanoplatforms can be selected for their detection, or biomarkers in cancer tissue and blood can be detected. In addition to identifying patients suitable for treatment with this, other means can also be used to make more patients suitable for the treatment with nanomaterials 119. For example, remodeling blood vessels in the TME may increase drug penetration. Subsequently, the patient's diagnosis should be made according to the specific treatment strategy, as cancer cells may be diagnosed with strong ferroptosis resistance. In general, ferroptosis-resistant cancer cells widely upregulate antioxidant defense systems, balancing with more intense intracellular oxidative stress. Currently, there is an urgent need to identify human-derived tumor-characteristic ferroptosis biomarkers in blood. However, further research and understanding. The purpose of diagnosis is to design treatment strategies for the synergy of ferroptosis with other treatment modalities. This requires targeted alteration of the patient's cancer tissue oxidative balance and remodeling of the TME.

At present, theranostics nanomaterials may be a good choice for clinical practice. Compared with traditional diagnostic nanoplatform, theranostics nanomaterials can be more precise in tracking and do not depend on the patient's blood or tissue samples. Theranostics nanomaterials also have the advantage of not only reducing costs in clinical translation, but also the complexity of treatment. Some nanomaterials with integrated diagnosis and treatment have been developed. It is recommended to develop theranostics nano prodrugs that signal "appropriate" treatment after remodeling the sensitivity of cells to ferroptosis and perform the subsequent treatment or synergize with other therapeutic strategies. For example, a theranostics nanomaterial prodrug with high ROS induction can increase intracellular ROS and "light up" cancer cells when accumulated to a certain level 120. Furthermore, theranostics nanomaterials that detect intracellular available iron levels can be developed or only target the level of lipid peroxidation. In addition, the utilization of GSH may be a good option for drugs that detect intracellular antioxidant activity.

### 5.2. Therapeutic strategy

Although FINs have exhibited significant induction of ferroptotic effects in the laboratory setting, unfortunately, no small molecule FINs have been approved for clinical treatment. Nevertheless, nanomaterials developed by using FINs are promising for clinical treatment. Detailed mechanisms regarding treatment have been explained in the above sections. Since the main purpose of developing nanomaterials is to improve treatment efficiency and reduce side effects, tumor penetration strategies with drugs, such as changing drug configuration (worm-like drugs 121, *etc*.), are worth considering. In addition, strategies should be taken to allow nanomaterials to be released at the right time, for example, using the low pH 122 of the tumor, hypoxic environment, *etc*. Alternatively, interventions, such as applying light, sound 123, and magnetic field, can be considered. Some ideas for therapy are proposed here.

Combining multiple drugs can reduce single toxicity, reduce the dosage of drugs, and enhance the therapeutic effect of drug-resistant tumors. For example, in ferroptosis-resistant cancer cells combining several drugs simultaneously weakens antioxidant defense systems and aggravates free iron. However, more clinical trials are needed for evaluating the effects of multidrug combinations on ferroptosis. In addition, one drug pleiotropic or multidrug release is also feasible, but aggravates the complexity in production and clinical application. Most importantly, a set of evaluation systems for nanomaterials that induce ferroptosis should be established to evaluate the efficacy of different model animals or therapeutic strategies. In addition, the changes of ferroptosis characteristics (such as iron, PUFAs, GSH) of tumors at different time points after administration have been determined and scored by algorithms to simulate the best efficacy. Also, different treatment strategies need to be selected for different types of immune microenvironments (already mentioned above). The effects of drugs on cancer cells and immune cells should be comprehensively considered. In addition, the combination of gene therapy and other nanomedicines targeting ferroptosis has also shown superiority. A recent study has well overcome ferroptosis tolerance in tumors and achieved durable curative effects by a combination of RNA interference and ferroptosis-target nanomedicines. Perhaps the combination of nanomedicines carrying ncRNAs and iron-based nanomedicines can have good clinical results. In summary, the development of more effective nanomedicine therapeutics targeting ferroptosis requires better imagination and understanding.

## 6. Conclusion and perspective

Ferroptosis, as a special type of programmed cell death modality, is a promising target in cancer treatment 124. Nanomaterial therapeutic strategies based on ferroptosis mechanism and small-molecule inhibitor construction continuously emerge. Although nanomaterials targeting ferroptosis have not yet been clinically approved, they have shown considerable promise. Meanwhile, with the increased understanding of the mechanism of ferroptosis, the diagnostic tools in this area are also continuously improving. However, researchers must fully consider the heterogeneity of the patient's TME. In the future, the general trend involves expanding new nanomaterials development ideas as well as precision-targeted ferroptosis therapy.

Although there have been exciting advances in the development of nanomaterials that induce ferroptosis, there are still problems that need to be urgently solved. The first is the problem of the limits of intracellular Fenton production. If the intracellular acidic environment is weak, the rate of ROS production dependent on hydrogen peroxide generation will be low. In terms of radiotherapy, how to achieve the efficient enrichment and clearance of nanomaterials in tumors has become the most important problem to be solved. In combination with immunotherapy, further understanding of the relationship between ferroptosis and immunity and TME is urgently needed to develop more precisely targeted nanomaterials. In addition, strategies need to be developed to guide the targeting of nanomaterials in patients. For instance, magnetic fields are used to guide the enrichment of nanomaterials that induce ferroptosis in tumors. Additionally, in the relationship between ferroptosis and antitumor immunity, more findings are needed to improve the understanding of ferroptosis in immune cells and cancer cells and further elucidate its differences 125. The development of nanomaterials that selectively target cells through differences between cells is key to promoting both ferroptosis and immunotherapy. In addition, researchers need to devote more attention to considering the clinical manifestations of nanomaterials that induce ferroptosis and establish an effective evaluation system. In summary, nanomaterials that induce ferroptosis have great potential, but there are still many aspects that need to be improved.

## Figures and Tables

**Table 1:** Representative nanomaterial-mediated ferroptosis

	Name	Mechanisms	Strategies to induce ferroptosis	Ref.
Iron	IO NPs	M1 macrophages release H_2_O_2_, which reacts with Fe^3+^ or Fe^2+^ to produce ROS *via* the Fenton reaction	Lipid peroxidation	74
	Cisplatin-loaded IO NPs	Used intracellular Fe^2+^ released from IO NPs to enhance sensitivity to cisplatin	Lipid peroxidation	77
	IO-LAHPNPs	Fe^2+^ is released from the surface of IO-LAHPNPs, which triggers the formation of ROS and O^2-^, leading to cancer cell death	Iron accumulation	78
	Assembled IO NPs	H_2_O_2_ is released and the Fenton reaction occurs, producing -OH	Lipid peroxidation	79
	(AFeNPs)	The Fenton reaction in tumors is induced using mild acidity and excess production of H_2_O_2_ in the TME	Iron accumulation, lipid peroxidation	87
	Iron-organic Frameworks	Fe^2+^ is released and induces the Fenton reaction, which increases the intracellular ROS concentration	Iron accumulation	74
	FePt NPs	It releases Fe^2+^, which can catalyze the breakdown of intracellular H_2_O_2_ into ROS	Iron accumulation	88
	Fe (Ⅲ)-ART (Artesunate) NPs	After the release of Fe^3+^, it is further reduced to Fe^2+^ catalyzes the endoperoxides of ART to generate C-centered radicals, leading to GSH depletion	Iron accumulation, antioxidant defence: GPX4 axis	89
	FeCO-DOX@MCN	Iron loading, ROS level increase, GSH depletion, GPX4 inactivation	Iron accumulation, antioxidant defence: GPX4 axis	90
	DGU:Fe/Dox	Dox release triggered by NIR (Near infrared radiation), iron loading, ROS accumulation, downregulation of GPX4 and ACSL4	Iron accumulation, antioxidant defence: GPX4 axis	91
	FeGd-HN@Pt@LF/ RGD2	Increase local concentrations of Fe^3+^, Fe^2+^ and H_2_O_2_ simultaneously	Iron accumulation	92
	SPFeN	Released Fe^3+^ is reduced to Fe^2+^, and • OH is generated by the Fenton reaction under acidic conditions	Iron accumulation	93
	FePt/MoS2	Killing of tumor cells by triggering a rapid Fenton reaction and photothermal therapy	Iron accumulation	94
	PYSNPs	Porous eggshell nanostructures of iron/Fe3O4 stabilize iron (0) and control the release of iron (0) in the TME and promote the Fenton reaction	Iron accumulation	95
	PEG-Fns	Monodisperse ferrate NPs, triggered by blue light at the tumor site generate Fe^2 +^	Iron accumulation	52
	SPION	Free iron species are released from the acidic environment of lysosomes, and the NIR photosensitizer Cy7-Hex anchors to the mitochondrial membrane where binding to sorafenib results in a burst of LPO (Lipid peroxidation)	Iron accumulation, antioxidant defence: GPX4 axis	96
	SRF@FeIIITA	SRFFeIIITA NPs can cause a corona dissociation reaction in response to the lysosomal acid environment, allowing the release of sorafenib to inhibit the GPX4 enzyme-triggered ferroptosis	Iron accumulation, antioxidant defence: GPX4 axis	97
	Mn-MOF	Continuously catalyzes the *in situ* generation of O_2_ from tumor-overexpressed H_2_O_2_, alleviates tumor hypoxia, decreases GSH and GPX4, and promotes the production of ROS and iron overload after ultrasound (US) irradiation in hypoxic tumors	Iron accumulation, antioxidant defence: GPX4 axis	98
	GBP@Fe3O4	triggered bylocalized moderate heat (45 °C), leading to burst release of Fe3O4 *in situ* to produce potent reactive oxygen species through the Fenton reaction in the tumor microenvironment	Lipid peroxidation	99
	DOX/Fe3+/EGCG (DF) NPs	The pH-corresponding nanomicelles, promote lipid peroxidation by releasing free iron and DOX	Iron accumulation, antioxidant defence: GPX4 axis	100
	bcc-USINPs	Strong Fenton response with good immunotherapeutic synergy	Iron accumulation	101
	PCGA@FeNP	Iron-based Nanomedicines Released by Photothermal Response	Iron accumulation	102
	FePPy NP	Killing cancer cells by enrichment of free iron and photothermal effects	Iron accumulation, lipid peroxidation	103
	CoFe2O4	Double Corresponding Fenton Reaction between sonodynamic therapy and chemodynamic therapy Triggers Nanomedicines	Lipid peroxidation	104
	Fe3O4-SAS@PLT	Platelet Membrane-Camouflaged Magnetic Nanoparticles, release iron and weaken antioxidant defenses	Iron accumulation, antioxidant defence: GPX4 axis	67
Non-iron	BCFe@SRF	In the hypoxic environment, BSA-Ce6 is released for laser-triggered PDT, ferritin is released for iron-catalyzed Fenton reaction, and SRF is released for tumor antioxidant defense system impairment	Strategies to induce ferroptosis	105
	ZnO NPs	Increases intracellular iron availability by affecting iron channels on mitochondria	Lipid peroxidation	106
	(US)-activatable nanomaterials	Impairment of antioxidant defense systems by released ferrate triggered by ultrasound overcomes the hypoxic environment	Lipid peroxidation	107
	Ce6@CMOF	Through photodynamic release, the disulfide-thiol exchange reaction leads to the depletion of intracellular GSH	Iron accumulation	85
	LDL-DHA	A low-density lipoprotein NP. The killing of cells by lipid peroxidation is triggered by the native omega-3 fatty acids	Lipid peroxidation	86
	mPEG-PLys-AA/RSL3	Lipid peroxidation products such as ROS can induce intracellular GSH failure and indirectly enhance the inhibitory effect of RSL3 on the GPX4 enzyme	Iron accumulation, lipid peroxidation	84
	miR-101-3p nanomaterials	Intracellular delivery of miR-101-3p restores ferroptosis in tumor cells by directly targeting TBLR1.	Iron accumulation	108
	SRF@Hb-Ce6	Photodynamic triggered nanomaterials, loaded sorafenib induces ferroptosis, and loaded heme promotes PDT and the Fenton reaction by oxygen release	Iron accumulation	45
	supramolecular Ce6-erastin nanodrug	Photodynamic triggered nanomaterials, loaded erastin leads to a decrease in system xc - and disrupt antioxidant defense systems	Iron accumulation, antioxidant defence: GPX4 axis	109
	HA-C60-Tf/AS	Targeting of Trf triggers ferroptosis in tumor cells through the loaded artemisinin	Iron accumulation, antioxidant defence: GPX4 axis	48
	FaPEG-MnMSN@SFB	Rapid clearance of GSH disrupts antioxidant defense systems by two mechanisms	Iron accumulation, antioxidant defence: GPX4 axis	110
	ZVI-NPs	Causes mitochondrial dysfunction, intracellular oxidative stress, and lipid peroxidation, promotes the degradation of Nrf2, leading to ferroptosis in cancer cells. It can also enhance macrophage M1 transformation and reduce PD-L1 expression in the TME.	Iron accumulation	68
	Pt-FMO	It has similar antitumor efficacy to cisplatin in targeting ferroptosis, but has lower toxicity.	Iron accumulation	111
	TMBF4TCNQ and TMB-TCNQ	Organic photothermal agent that absorbs near-infrared light, effectively inhibits the intracellular biosynthesis of GSH, leading to redox stress and ROS-mediated ferroptosis	Iron accumulation	112
	PBE	Ferroptosis nanomaterials, triggered by acid-base changes, release RSL3 impairs antioxidant defense systems under acidic conditions and can synergize with immunotherapy.	Iron accumulation	69
	Fe3O4-SAS @ PLT	Triggers ferroptosis through the loaded SAS and shows good synergistic immunotherapeutic effects	Iron accumulation	67
	RSL3 @ COF-Fc(2b)	Induces ferroptosis by suppressing antioxidant defense systems and generating oxygen radicals	Iron accumulation, antioxidant defence: GPX4 axis	113
	MnO2@HMCu_2-x_S	Photothermal triggering, release of manganese ions promotes lipid peroxidation, and mediates autophagy to aid in the development of ferroptosis	Lipid peroxidation	114
	GOx/BSO@CS PVs	Treatment of Cancer by Induction of Iron Death Synergistic Hunger Therapy	Lipid peroxidation	115
	FeOOH NSs	Imageable nanomedicines that alter the cellular oxidative environment by producing hydrogen sulfide	Lipid peroxidation	116
	amorphous calcium phosphate (ACP)-based nanoplatform	Multi-purpose combined targeted therapy nanoplatforms	Lipid peroxidation	117

## Abbreviations

ACSL3: Acyl-CoA Synthetase Long Chain Family Member 3
ACSL4: Acyl-CoA Synthetase Long Chain Family Member 4
ART: Artesunate
ATM: Ataxia Telangiectasia Mutated
CYB5R1: Cytochrome B5 Reductase 1
DAMP: Damage-associated Molecular Pattern
DHODH: Dihydroorotate Dehydrogenase (Quinone)
EMT: Epithelial-mesenchymal Transition
FDA: Food and Drug Administration
FMN: Flavin Mononucleotide
FPN1/SLC40A1: ferroportin 1/ solute carrier family 40 member 1
FSP1: ferroptosis suppressor protein 1
FXN: Frataxin
GCLC: Glutamate-Cysteine Ligase Catalytic Subunit
GPX4: Glutathione Peroxidase 4
GSH: Glutathione
HIFs: Hypoxia Inducible Factors
KRAS: KRAS proto-oncogene, GTPase
LIP: Labile Iron Pool
LPCAT3: Lysophosphatidylcholine Acyltransferase 3
LPO: Lipid peroxidation
NIR: Near infrared radiation
NRF2: NFE2-Related Factor 2
NTBI: non-transferrin-bound iron
PD-L1 (CD274): Programmed Death Ligand 1
PCD: Programmed cell death
POR: Cytochrome P450 Oxidoreductase
PUFA/MUFA: polyunsaturated fatty acid/ monounsaturated fatty acids
ROS: Reactive Oxygen Species
SCARA5: Scavenger Receptor Class A Member 5
SLC11A2: Solute Carrier Family 11 Member 2
SLC39A14: Solute Carrier Family 39 Member 14
SLC39A8: Solute Carrier Family 39 Member 8
SLC3A2: Solute Carrier Family 3 Member 2
SLC7A11: Solute Carrier Family 7 Member 11
TfR1: Transferrin Receptor 1
TME: Tumor microenvironment
